# Comparing Effects of BK Virus Agnoprotein and Herpes Simplex-1 ICP47 on MHC-I and MHC-II Expression

**DOI:** 10.1155/2013/626823

**Published:** 2013-03-27

**Authors:** Michela Cioni, Christian Mittelholzer, Marion Wernli, Hans H. Hirsch

**Affiliations:** ^1^Transplantation & Clinical Virology, Department Biomedicine Haus Petersplatz, University of Basel, Petersplatz 10, CH-4003 Basel, Switzerland; ^2^Infectious Diseases & Hospital Epidemiology, University Hospital Basel, Petersgraben 4, CH-4031 Basel, Switzerland

## Abstract

*Background*. Among human polyomaviruses, only BK virus (BKV) and JC virus (JCV) encode an agnoprotein upstream of VP1 on the viral late transcript. BKV agnoprotein is abundantly expressed late in the viral life cycle, but specific cellular and humoral immune responses are low or absent. We hypothesized that agnoprotein might contribute to BKV immune evasion by downregulating HLA expression, similar to Herpes simplex virus-1 ICP47. Methods UTA-6 or primary human renal proximal tubular epithelial cells (RPTEC) were co-transfected with plasmids constitutively expressing agnoprotein, or ICP47, and enhanced green-fluorescent protein (EGFP). EGFP-gated cells were analyzed for HLA-ABC and HLA-DR expression by flow cytometry. HLA-ABC and HLA-DR expression was also analyzed on UTA-6 bearing tetracycline-regulated agnoprotein or ICP47. Effects of agnoprotein on viral peptide-dependent T-cell killing were investigated using ^51^Cr release. *Results*. ICP47 downregulated HLA-ABC without affecting HLA-DR, whereas agnoprotein did not affect HLA-ABC or HLA-DR expression. Interferon-**γ** treatment increased HLA-ABC in a dose-dependent manner, which was antagonized by ICP47, but not by agnoprotein. In UTA-6 cells, agnoprotein expression did neither impair HLA-ABC or -DR expression nor peptide-specific killing impaired by HLA-matched T-cells. *Conclusion*. Unlike the HSV-1 ICP47, BKV agnoprotein does not contribute to viral immune evasion by down-regulating HLA-ABC, or interfere with HLA-DR expression or peptide-dependent T-cell cytotoxicity.

## 1. Introduction

Human polyomavirus (HPyV) species comprise currently at least 9 members that infect 30 to 90 percent of the general population without severe clinical manifestations [1–6]. In immunocompromised individuals, however, significant pathologies have been linked to HPyVs, for example, nephropathy and haemorrhagic cystitis to BKV, progressive multifocal leukoencephalopathy to JCV, *trichodysplasia spinulosa* to TSPyV and Merkel cell carcinoma to MCPyV [7, 8]. The general architecture of the approximately 5.1 kb circular dsDNA genome is shared among all HPyVs consisting of the noncoding control region separating the early nonstructural genes encoding small and large T antigen and late capsid VP1, -2, and -3 genes [6]. So far, however, only BKV and JCV contain an additional small conserved open reading frame (ORF) in the late gene transcript upstream of the major capsid protein VP1 [9, 10], a feature shared by the simian virus SV40, but not by other HPyVs [11]. The encoded protein of 60–70 amino acids (aa) has been termed agnoprotein, and, despite multiple studies, its major function in the biology of BKV or JCV has not been identified [12–14]. Agnoprotein is abundantly expressed late in the viral life cycle, and localizes predominantly to the cytoplasm both *in vitro* [9, 15] and also *in vivo* as shown in biopsies of BKV-associated nephropathy [16]. In the cytoplasm, BKV agnoprotein shows a reticular distribution but also colocalizes with lipid droplets, the latter being mediated by an amphipathic helix of 20 aa in the central part of the 66 aa BKV agnoprotein [15]. 

Despite its abundant expression of BKV agnoprotein *in vivo*, agnoprotein-specific humoral and cellular immune responses were low or undetectable in individuals exposed to BKV [16]. This observation was contrasted by the strong immune responses found for the BKV capsid protein VP1 located on the same transcript or for the amino-terminal domain shared between small and large T antigen [16–18]. Since BKV and JCV persist lifelong after primary infection and are asymptomatically shed in immunocompetent healthy individuals [3], we speculated that agnoprotein might have a role in immune evasion. Interestingly, histopathology studies reported decreased MHC-II expression of BKV-infected renal tubular epithelial cells as a potential marker distinguishing BKV-associated nephropathy from acute cellular rejection [19].

Multiple mechanisms of viral immune evasion have been described. The immediate early protein ICP47 of Herpes simplex virus-1 (HSV-1) has been reported to downregulate HLA class-I antigen presentation by specifically binding to and blocking TAP, the transporter associated with intracellular export of peptides generated from intracellular proteins by the proteasome. Thereby, peptide epitope loading and presentation of MHC-peptide complexes on the cell surface is impaired and decreases immune recognition by cytotoxic T lymphocytes [20]. HSV-ICP47 is a small protein of 88 amino acids, its functional domain maps at the N-terminus, and the secondary structure is predicted to contain amphipathic helix structures [21, 22]. We therefore hypothesized that the BKV agnoprotein might have a similar immunomodulatory role and investigated its effects on HLA-ABC and HLA-DR surface expression in transiently and stably transfected cells.

## 2. Materials and Methods

### 2.1. Plasmids

The pCMV-agno plasmid was a kind gift from Dr. Christine Rinaldo, Tromsø, Norway [9]. The hygromycin selectable expression plasmid phTRE-agno was generated by subcloning the agnoprotein coding sequence from pTRE-agno into pTRE2hyg using BamHI and NotI restriction sites. The pCMV-ICP47 and phTRE-ICP47 were generated by replacing the agnoprotein coding sequence with the corresponding sequence encoding ICP47 (a kind gift from Dr. Paul Zajac, University Hospital of Basel, Switzerland). pEGFP-N1 was purchased from BD Biosciences.

### 2.2. Transfection

Cells were transfected with GeneExpresso 8000 (IV-1047; Lab Supply Mall, Inno-Vita Inc., Gaithersburg, MD,USA), according to manufacturers instructions. Briefly, for a well of a 6-well plate, cells were seeded in 1.5 mL of culture medium; 3 *μ*L of transfection reagent were diluted in 250 *μ*L of OptiMEM (Gibco) and kept for 5 minutes at RT; 4 *μ*g of DNA were diluted in 250 *μ*L of OptiMEM, and then mixed with the diluted transfection reagent for 20 minutes at room temperature before dropwise adding to the cells. After 4 hours of incubation at 37°C and 5%  CO_2_, medium was replaced. UTA-6 cells were cultured in presence of Tet and G418, unless indicated otherwise.

### 2.3. Cells

UTA-6 cells [23] were maintained in high glucose Dulbecco's Modified Eagle's Medium (DMEM, Sigma) with 10% Fetal Bovine Serum (FBS, Biochrome AG, Berlin, Germany), 2 mM L-glutamine, supplemented with 500 *μ*g/mL G418 (Geneticin, Sigma) and 1 *μ*g/mL tetracycline (Tet) (Sigma). For inducible protein expression experiments, medium containing 1 *μ*g/mL Tet was replaced with medium without Tet for 48 hours.

Primary human renal proximal tubular epithelial cells (RPTEC; ATCC PCS-400-010, lot 5321) were maintained in epithelial cell medium (EpiCM, ScienCell, Carlsbad, CA, USA) and passaged with Passage Kit 2 (2040002, Provitro GmbH, Berlin, Germany).

UTA-6 agno clones stably expressing the BKV agnoprotein in a Tet-dependent manner were obtained by cotransfecting the hygromcyin-selectable BKV agnoprotein-encoding construct phTRE-Agno and the EGFP encoding construct pEGFP-N1 (Clontech, Mountain View, CA, USA) in a 10 : 1 ratio. One day after transfection, cells expressing EGFP were sorted using a cell sorter (FACSARIA, BD) and seeded in 96 multiwell plates at a limiting dilution of 0.3 cells per 100 *μ*L of DMEM containing 10% FCS supplemented with 500 *μ*g/mL G418 (Geneticin, Sigma), 1 *μ*g/mL Tet (Sigma) and 800 *μ*g/mL hygromycin-B (Calbiochem). Medium was changed every 48 hours for 14 days to select clones. Subsequently the clones were expanded in medium containing 500 *μ*g/mL G418, 1 *μ*g/mL Tet and 200 *μ*g/mL hygromycin-B, tested by immunofluorescence for BKV agnoprotein expression after Tet removal. Clones with a Tet-dependent BKV agnroprotein expression were cryopreserved.

### 2.4. Interferon-*γ* Treatment

Recombinant human interferon-*γ* (IFN*γ*) was purchased from Peprotech (Rocky Hill, NJ, USA) and diluted in the cell medium at 100 U/mL, 10 U/mL and 1 U/mL. UTA-6 cells (4 × 10^4^) or RPTECs (1 × 10^5^) were seeded in a 24 multiwell plate. Serial dilutions of IFN*γ* were added to the culture medium and left at 37°C, 5% CO_2_ for 48 hours.

### 2.5. Antiagnoprotein Rabbit Serum

To detect expression of BKV agnoprotein or HSV ICP47, the following antibodies were used: antiagnoprotein rabbit serum (a kind gift from Dr. Christine Rinaldo, Tromsø, Norway) diluted 1 : 800 and anti-ICP47 rabbit serum (a kind gift from Dr. Klaus Frueh, Portland, ON, USA) diluted 1 : 750 in 3% milk/PBS. Cells were seeded onto glass coverslips and, after 2 days, washed with phosphate-buffered saline w/o Ca^2+^ and Mg^2+^ (PBS) and fixed using 4% paraformaldehyde at room temperature for 20 minutes. After two rounds of washing with PBS, the cell membranes were permeabilized with 0.2% Triton at room temperature for 10 minutes, washed again with PBS, and blocked for 15 minutes at 37°C using 3% milk/PBS. Coverslips were then stained with primary antibodies for 60 minutes at 37°C and washed three times in PBS. Then, Hoechst 33342 (0.5 *μ*g/mL; H21492, Invitrogen) and fluorescently labeled secondary antibody (anti-rabbit-Cy3 1 : 2000, 111-165-144, Jackson Immunoresearch, West Grove, PA, USA) were added and incubated for 60 minutes at 37°C. After 3 washes in PBS, the slides were mounted in 90% Glycerol (1.04095, Merck, Darmstadt, Germany) in PBS containing 1% *N*-propyl gallate (P-3130, Sigma) as an antifading agent.

### 2.6. HLA Quantitation by FACS

Cells were harvested and 5 × 10^4^ were resuspended in 50 *μ*L of washing buffer ( 0.5% BSA/PBS) and stained with 10 *μ*L of antibody anti-HLA-ABC PE conjugate and 5 *μ*L of anti-HLA-DR APC conjugate (Becton Dickinson). Samples were incubated for 30 minutes at 4°C in the dark, then washed twice with washing buffer, and centrifuged at 400 ×g for 5 minutes at 4°C. Supernatant was discarded, and cells were resuspended in 400 *μ*L of washing buffer and analyzed using the FACS Canto (Becton Dickinson Franklin Lakes, NJ, USA) and FACS Diva or Flow Jo software.

### 2.7. Peripheral Blood Mononuclear Cells Recovery and *In Vitro *Stimulation

Peripheral blood mononuclear cells** (**PBMCs) were obtained from two healthy donors, who gave written informed consent to the protocol (IRB 267/06) approved by the local institutional review board. Blood samples were diluted 1 : 2 in PBS, overlaid on Ficoll (Lymphoprep, Axis-Shield PoC AS, Oslo, Norway), and centrifuged at 800 ×g for 25 minutes at RT. The PBMCs band was recovered and washed twice at 300 ×g for 10 minutes at RT in PBS, and cells were then counted and resuspended in RPMI-1640 (Sigma-Aldrich Chemie GmbH Buchs SG, Switzerland) supplemented with 5% Human Serum AB (Sigma) and 2 mM of L-Ala-Glutamine termed R5. PBMCs were seeded in a concentration of 6 × 10^6^ cells per well in 3 mL of R5 medium in 6-well plates and incubated overnight at 37°C and 5% CO_2_. Floating cells were resuspended at a concentration of 2 × 10^6^ in 500 *μ*L of R5 in wells of 24 multiwell plate. Adherent cells were scraped, and the viable ones counted with Trypan Blue solution and resuspended at the concentration of 4 × 10^6^ per 1 mL in R5. After incubation with 5 *μ*g/mL of peptide for antigenic stimulation for 2 h at 37°C 5% CO_2_, the adherent cells were added to the floating cells in a 1 : 10 ratio. Incubation was continued for 5 to 12 days prior to testing. The peptides were derived from cytomegalovirus (CMV)-pp65 consisting of a 15-mer peptide (AGILARNLVPMVATV), a 9-mer peptide (NLVPMVATV) and a pool of peptides of 15-mer length overlapping for 11 aa length spanning the entire open reading frame of CMV-pp65 (Eurogentec Deutschland GmbH, Köln, Germany).

### 2.8. Interferon-*γ* EliSpot Assay (ESA)

PDVF multiscreen 96-well filter plates (MSIPS4W10, Millipore Bedford, MA, USA) were coated with 100 *μ*L of anti-IFN*γ* mAb 1-D1 K (Mabtech, Nacka, Sweden) at 10 *μ*g/mL and incubated overnight at 4°C. *In vitro* stimulated T cells were seeded at 1 × 10^5^/well in presence of 2 *μ*g/mL of peptide. Cells without any peptide were used as negative control, whereas cells stimulated with phytohemagglutinin (PHA; Sigma-Aldrich Chemie GmbH Buchs SG, Switzerland) were used as positive control. After incubation for 24 h at 37°C, anti-IFN*γ* mAb 7-B6-1-Biotin (Mabtech, Nacka, Sweden) at 1 *μ*g/mL was added for 2 h at RT, then Streptavidin ALP (Mabtech, Nacka, Sweden) at 1 *μ*g/mL for 1 h at RT, and SigmaFast BCIP/NBT (Sigma-Aldrich Chemie GmbH Buchs SG, Switzerland) for 20 minutes at RT in the dark. Plates were rinsed with water and dried, and spots were counted with an EliSpot reader (Cellular Technology Ltd Europe, Bonn, Germany). 

### 2.9. Killing Assay

Specific cytotoxic activity of *in vitro* stimulated T cells on UTA-6 cells was assessed by ^51^Cr release assay. Target cells were cultured for 24 hours prior to testing in presence or absence of Tet, labeled for 1 h at 37°C with 250 *μ*Ci per 1 × 10^6^ cells with ^51^Cr (Sodium Chromate Hartmann Analytic, Braunschweig, Germany) and then pulsed for 1 h at 37°C with 2 *μ*g/mL with CMV-pp65 peptides or without as negative control. The stimulated T cells were incubated with 5000 target cells at an effector: target (E : T) ratios of 80 : 1, 40 : 1, 20 : 1, 10 : 1, 5 : 1, and 2.5 : 1 at 37°C and 5%  CO_2_ for 4 h, and then 50 *μ*L of the supernatants were transferred to a lumaplate (Perkin Elmer, Waltham, MA, USA), dried and counts per minutes (cpm) were counted in a *β*-counter (TopCount, Perking Elmer). Killing assay data are reported as average of duplicate wells and as percentage of lysis, which is calculated according to the following formula: (Sample cpm-Spontaneous Release cpm) / (Maximum Release cpm / Spontaneous Release cpm) × 100, where the spontaneous release corresponds to the ^51^Cr released by the target cells alone and the maximum release corresponds to the ^51^Cr released by the target cells lysed with 2.5% SDS. Percentage of specific lysis was obtained subtracting percentage of lysis of the negative control. Data were considered reliable when the minimum release was less than 50% of the maximum release.

## 3. Results

**Figure 1:**
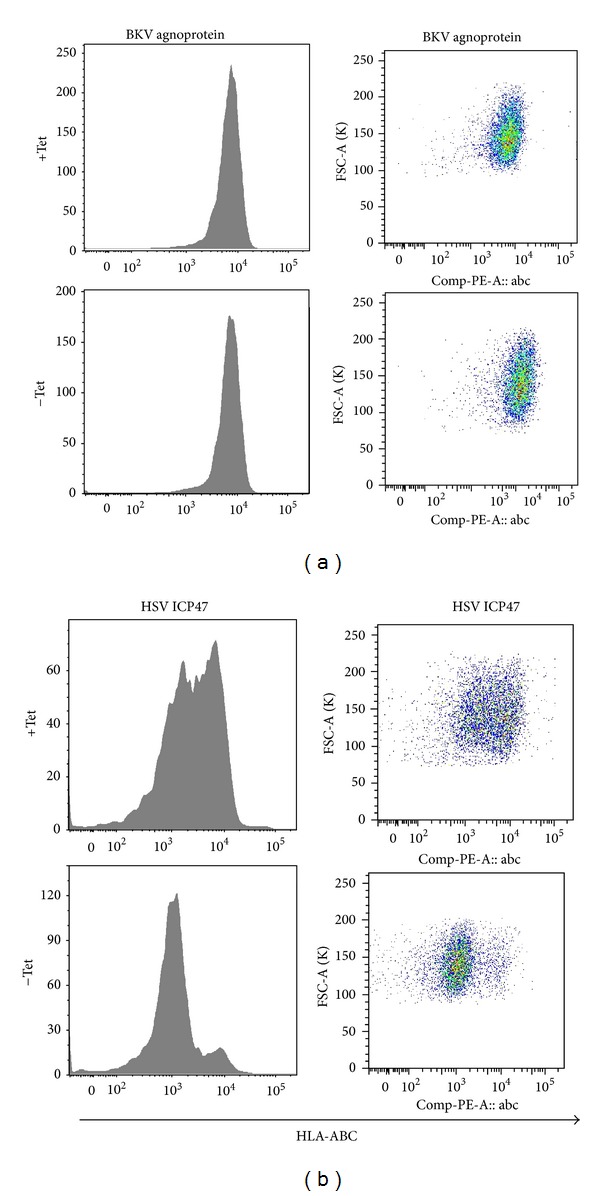
HLA-ABC cell surface expression in transfected UTA-6. Cells were cotransfected with pEGFP-N1 and the Tet-off regulated phTRE-agno or pTRE-ICP47 at a 1 : 10 ratio. HLA-ABC expression was analyzed by flow cytometry using labeled antibodies on EGFP-gated UTA-6 cells 48 h after transfection and 24 h after Tet-off induction.

To investigate the effect of the BKV agnoprotein on MHC class-I and class-II expression, we transfected UTA-6 cells with plasmids containing BKV agno or HSV-1 ICP47 under the control of a Tetracycline-(Tet-) off regulated promoter. Both expression constructs were cotransfected in 10-fold excess to an EGFP expression vector to permit enriching for transfected cells. In fixed cells, prominent expression of BKV agno or ICP47 could be seen after 24 and 48 hours following Tet removal, whereas expression was suppressed in the presence of Tet (not shown). To enumerate HLA class-I and class-II expression in transfected cells, the cells were stained using anti-HLA-ABC and anti-HLA-DR antibodies and the corresponding signals on EGFP-gated cells were quantified by flow cytometry. As shown, the removal of Tet was associated with a significant downregulation of HLA-ABC molecules in the Tet-regulated ICP47 transfectants, whereas no effect on HLA-ABC expression was seen for the Tet-regulated BKV agno transfectants (Figure 1).

**Figure 2:**
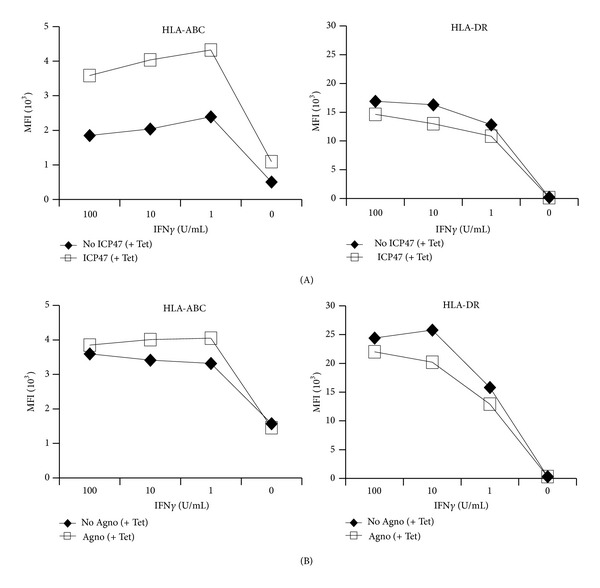
(A) HLA-ABC and -DR surface expression in UTA-6 cells transiently transfected with ICP47. HLA-ABC and -DR surface expression was analyzed by flow cytometry of EGFP-gated UTA-6 cells, cotransfected with pEGFP-N1 and pTRE-ICP47, and cultured for 48 hours with different concentration of IFN*γ*, with (□) or without (*♦*) tetracycline. MFI: mean fluorescence intensity. (B) HLA-ABC and -DR surface expression in UTA-6 cells transiently transfected with agnoprotein. HLA-ABC and -DR surface expression was analyzed by flow cytometry of EGFP-gated UTA-6 cells, cotransfected with pEGFP-N1 and phTRE-agno, and cultured for 48 hours with different concentration of IFN*γ*, with (□) or without (*♦*) tetracycline. MFI: mean fluorescence intensity.

To investigate the possibility that BKV agno elicited its function more prominently under circumstances of inflammation and immune activation such as interferon-*γ* (IFN*γ*) exposure, we treated transiently transfected UTA-6 cells with increasing concentrations of IFN*γ* known to increase MHC-expression. Indeed, significant upregulation of HLA-ABC expression was seen in response to 1 U/mL IFN*γ* with little further increases in response to 10 or 100 U/mL IFN*γ* (Figure 2). This IFN*γ* -induced HLA-ABC upregulation was clearly reduced in HSV-1 ICP47-expressing cells (Figure 2(A)) but not affected by BKV agno expression (Figure 2(B)). For HLA-DR expression, a significant increase in response to IFN*γ* became also apparent but was not affected by BKV agno- or by ICP47-expression (Figures 2(A) and 2(B)). Together, the results indicated that dynamic changes in HLA-expression could be monitored by flow cytometry as indicated by the responses to IFN*γ*. HLA-ABC or HLA-DR expression was not significantly affected by BKV agnoprotein, with or without IFN*γ*-mediated upregulation. By contrast, HSV-1 ICP47 selectively interfered with HLA-ABC expression but not with HLA-DR. 

**Figure 3:**
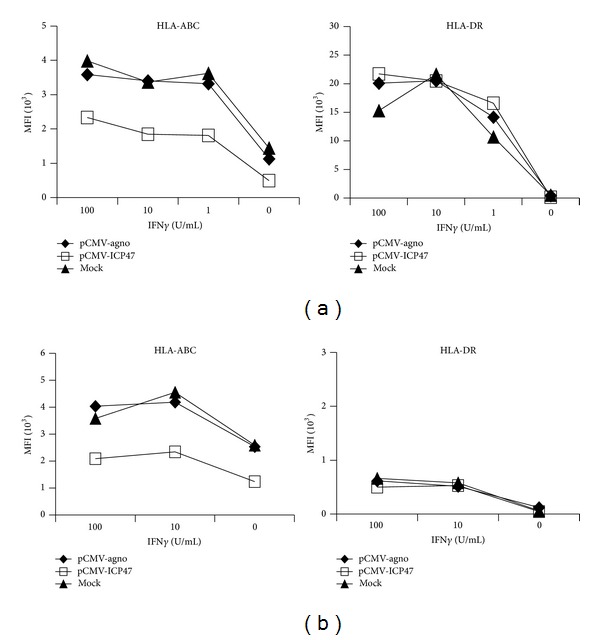
(a) HLA-ABC and -DR surface expression in transiently transfected RPTECs. Cells were cotransfected with pEGFP-N1 and either pCMV-agno or pCMV-ICP47 at a 1 : 10 ratio. pEGFP-N1 alone (mock, ▲) or together with pCMV-agno (*♦*) or with pCMV-ICP47 (□) as described above. MFI: mean fluorescence intensity. (b) HLA-ABC and -DR surface expression in transiently transfected UTA-6. UTA-6 cells were cotransfected with pEGFP-N1 and either pCMV-agno or pCMV-ICP47 at a 1 : 10 ratio. pEGFP-N1 alone (mock, ▲) or together with pCMV-agno (*♦*) or with pCMV-ICP47 (□) as described above. MFI: mean fluorescence intensity.

To examine the effects of BKV agno and HSV ICP47 on HLA class-I and class-II expression of RPTECs (Figure 3), the corresponding constructs under the control of the constitutive CMV promoter were cotransfected with the EGFP plasmid in a 10 : 1 ratio. HLA-ABC and HLA-DR expression was evaluated in EGFP-gated cells by flow cytometry at 24 h with or without IFN*γ* stimulation. As shown (Figure 3(a)), IFN*γ* treatment significantly increased HLA-ABC expression but had only minor effects on HLA-DR expression. HLA-ABC expression was reduced in ICP47-transfected RPTECs, whereas HLA-DR expression levels were not significantly affected. By contrast, agnoprotein-transfected cells had no discernible effect on HLA-ABC or HLA-DR expression. Similar results were obtained when UTA-6 cells were cotransfected with either the constitutive CMV-driven BKV agnoprotein or HSV-1 ICP47 together with EGFP (Figure 3(b)).

**Figure 4:**
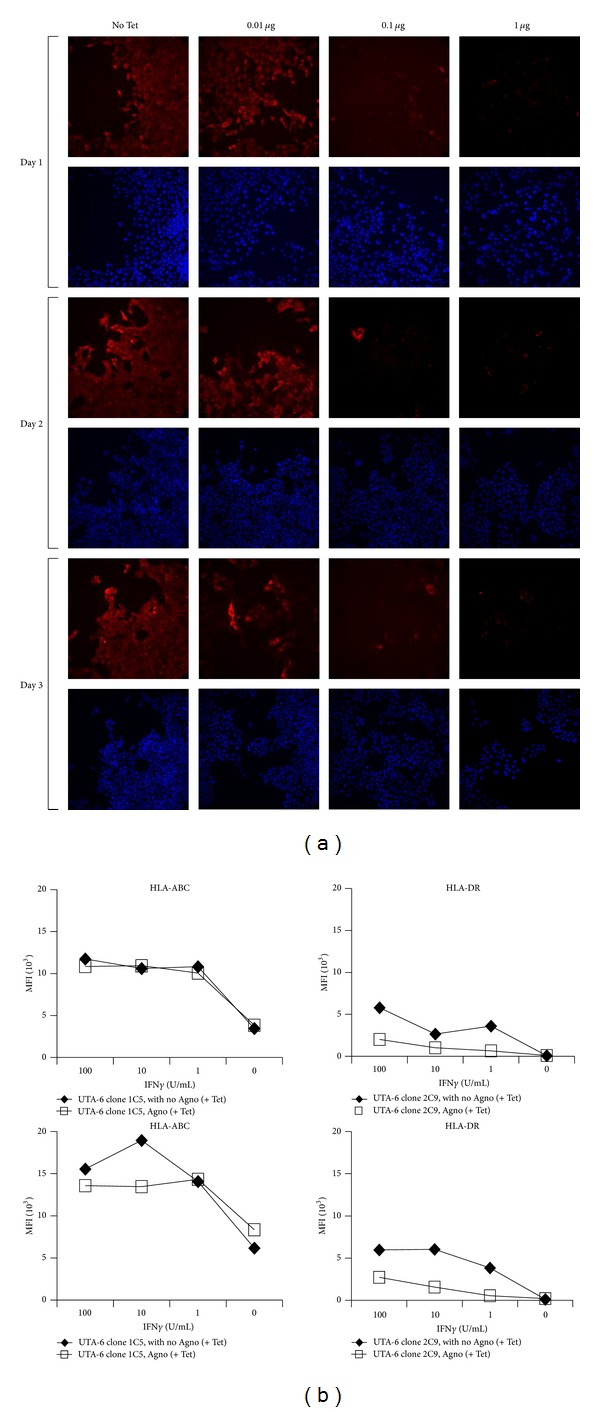
(a) Regulation of BKV agnoprotein expression in the stably transfected UTA-6 clone 2C9. The panel shows a time course experiment using different concentrations of tetracycline. Blue: DNA stained with Hoechst 3342. Red: agnoprotein detected by indirect immunofluorescence. (b) HLA-ABC and -DR surface expression in UTA-6 clonal cells with Tet-off inducible agnoprotein expression. HLA-ABC and -DR surface expression was detected by flow cytometry in the UTA-6 clone 1C5 (first row) and 2C9 (second row) and cultured for 48 hours with different concentration of IFN*γ* and for 24 h in the presence (□) or absence (*♦*) of Tet. MFI: mean fluorescence intensity.

To verify that any potential effect of BKV agno expression could be compared in the same cell population, we analysed selected UTA-6 cell clones that were stably transfected with a Tet-regulated construct and expressed agno in a tightly regulated manner. BKV agno was expressed at different levels depending on the Tet concentrations (Figure 4(a)). After 48 hours of incubation with or without IFN*γ*, HLA-ABC expression was not significantly altered in the presence or absence of agno expression, and if at all, a slight increase in HLA-DR expression was seen after induction in UTA-6 cells clones (Figure 4(b)).

**Figure 5:**
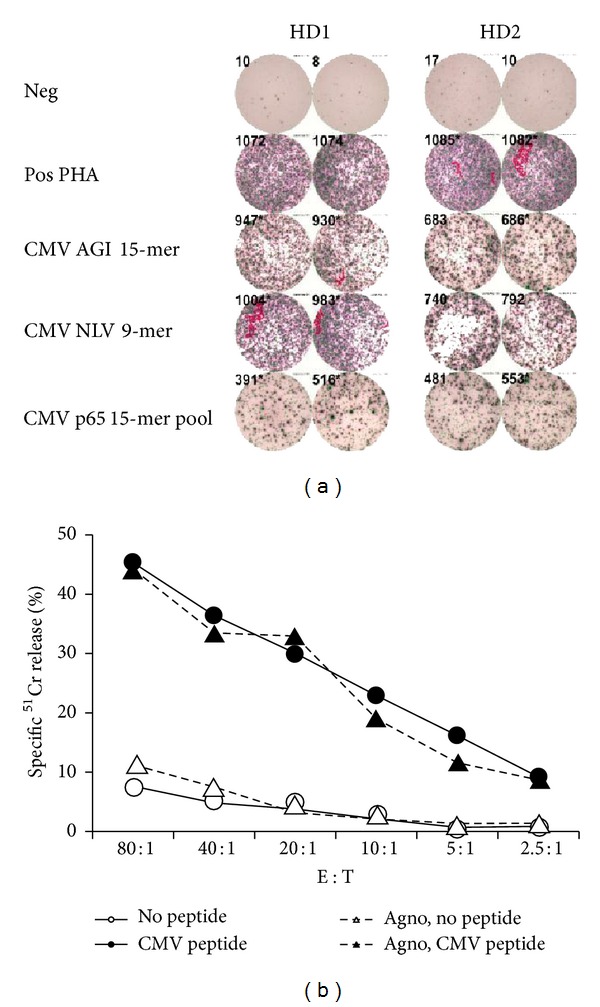
(a) IFN*γ* production by CMVpp65-specific T cells: IFN*γ* SFUs are shown for 1 × 10^5^ T cells from healthy donor 1 (HD1) and 2 (HD2) challenged with medium (neg), phytohemagglutinin (PHA), CMV-pp65-15 m123, and CMV-pp65-9 m495, CMV-pp65-15 Mp. (b) Effect of Tet-off inducible BKV agnoprotein expression on CTL activity. UTA-6 clone 2C9 cells were cultured for 24 h in the presence of Tet (continuous line) or absence of Tet expressing agnoprotein (dotted line, agno), labeled with ^51^Cr and then pulsed without (△, ◯ ) or with (▲, ◯ ) CMV 9-mer peptide NLVPMVATV. CMV antigen-specific T cells from a healthy donor was added in the indicated effector : target ratios and percent release of  ^51^Cr measured.

We next investigated the potential effect of BKV agnoprotein on HLA-peptide-dependent killing by antigen-specific cytotoxic T lymphocytes (CTL). The HLA-0201 positive UTA-6 cells expressing agnoprotein under Tet-off regulation were used as targets and HLA-A0201 specific T cells were used as effectors in a ^51^Cr release assay. Peripheral blood mononuclear cells (PBMCs) from two HLA-A0201 healthy donors (HD1 and HD2) were stimulated with a 15-mer peptide derived from CMV-pp65 (AGILARNLVPMVATV) containing the well-characterized HLA-A0201 restricted immunodominant nonameric sequence NLVPMVATV. The EliSpot assay of the HD1 and HD2 cell preparations showed a peptide-specific IFN-*γ* response with the 15-mer and with the CD8-specific CMV 9-mer NLVPMVATV (Figure 5(a)). The cells were added to ^51^Cr-labeled UTA-6 cells pulsed with CMV 9-mer NLVPMVATV 24 hours after inducing or not inducing BKV agnoprotein (Figure 5(b)). The results of ^51^Cr release assay showed that CTL of HD1 could efficiently kill UTA-6 cells in an E : T- dependent ratio, which was not affected by agnoprotein expression (Figure 5(b)). Similar results were obtained with HLA A-0201 matched T cells obtained from HD2 (not shown). The data demonstrated that agnoprotein had no influence on HLA-0201—peptide-dependent killing by cytotoxic T cells.

## 4. Discussion

Immune evasion has been recognized as an important feature of viruses to subvert innate and adaptive immune responses [24]. Frequently, different steps of the MHC-I peptide loading and surface presentation are targeted in line with the notion that impaired recognition of infected cells by virus-specific cytotoxic CD8 T cells must provide significant advantages. Among persisting human DNA viruses, the adenovirus E3-19 K and the cytomegalovirus U3 proteins were shown to interfere with epitope peptide loading via the peptide transporter TAP and tapasin, while the cytomegalovirus US6 protein prevents transport of peptide epitopes to the endoplasmic reticulum side causing MHC-I retention and degradation. On the other hand, the HSV-1 ICP47 causes the decrease in MHC-I surface molecules by blocking peptide transport through binding to TAP on its cytoplasmic side and thereby decreasing immune recognition by cytotoxic T-lymphocytes [20]. BKV and JCV can be regarded as viruses successfully adapted to the human host [25] given the observation that 50 percent to 90 percent of the world's population is infected [3, 5] and 10–40 percent of healthy immunocompetent adults continue to shed these viruses asymptomatically in the urine [3]. These high rates of infection and asymptomatic replication together with the fact that significant BKV and JCV diseases are almost exclusively seen in immunodeficient patients suggest that some immune control is operative in healthy individuals but not to an extent that permits complete suppression and/or elimination from the infected host. Since both BKV and JCV express a highly homologous agnoprotein, a potential role in immune evasion could be hypothesized. We observed that there are some structural similarities between HSV-ICP47 and BKV agnoprotein, as they are both small cytoplasmic proteins, bearing a central domain of an amphipathic helix. Given these similarities and the fact that HSV-ICP47 has been shown to downregulate MHC-I expression, we hypothesized that BKV agnoprotein could play a similar role, contributing to BKV immune evasion. Moreover, it was previously reported that BKV agnoprotein could negatively influence the transport of a temperature-sensitive VSV virus glycoprotein to the cell surface [12]. MHC-I molecules stabilized by loaded peptides are continuously transported from the endoplasmic reticulum to the cell surface for immune display. The results reported here demonstrated that expression of BKV agnoprotein had no discernible influence on HLA-ABC or HLA-DR. Stimulation with interferon-*γ* resulted in an increase in HLA-ABC expression indicating that relevant quantitative increases in surface HLA-ABC levels could be detected by flowcytometry. Expression of the HSV-1 ICP47 effectively downregulated this interferon-*γ* response of HLA-ABC, but had no effect on the HLA-DR upregulation in line with the specificity of this effect. By contrast, BKV agnoprotein showed no effect on HLA-ABC or HLA-DR expression, with or without prior interferon-*γ* stimulation in UTA-6 as well as in RPTECs as the natural host cell target of BKV. The data suggest that BKV agnoprotein does not exert a similar effect on immune evasion as HSV-ICP47. The data are of interest, since clinical pathology data of kidney allograft biopsies suggested that HLA-DR might be reduced in interstitial nephritis due to BKV-associated nephropathy as compared to those showing T cell-mediated interstitial rejection [19].

Results obtained in transiently transfected cells have different limitations, in terms of efficiency of transfection and durability of protein expression. However, our earlier studies on BKV agnoprotein suggested that under these circumstances, infection and transfection provide comparable results regarding the magnitude and the subcellular localization of BKV agnoprotein [15]. The response of stable, inducible clones confirmed the observations in transiently transfected cells. Importantly, under these conditions, HSV-1 ICP47 was able to elicit its specific downregulating effects on HLA-ABC rendering a severe limitation of our approach unlikely.

As the HLA-ABC and -DR molecules were not numerically reduced on the cell surface, we investigated the possibility of a functional impairment mediated by BKV agnoprotein. To test this hypothesis, we used a well-characterized, immunodominant 9-mer peptide from the CMV-pp65 antigen, which is an immunodominant target of cytotoxic T cell response *in vitro* and *in vivo* in HLA-A0201 positive individuals. As shown, BKV agnoprotein expression failed to interfere with CMV 9-mer NLVPMVATV-dependent killing arguing against a major role of BKV agnoprotein in modulating a functional immune evasion of this kind. In conclusion, we demonstrate that BKV agnoprotein does not modulate surface expression of HLA-ABC and -DR or interfere with HLA-A0201-mediated CTL activity. Thus, other functions and mechanisms of BKV agnoprotein need to be considered which may or may not include a role in immune evasion.
